# Concomitant medications and circulating tumor cells: friends or foes?

**DOI:** 10.20517/cdr.2022.68

**Published:** 2023-01-04

**Authors:** Serena Di Cosimo, Vera Cappelletti

**Affiliations:** Department of Advanced Diagnostics, Fondazione IRCCS Istituto Nazionale dei Tumori, Milano 20133, Italy.

**Keywords:** Breast cancer, concomitant medications, circulating tumor cells

## Abstract

The use of concomitant medications by patients with cancer is observed almost globally; however, little attention has been paid to this topic in the medical literature. Most clinical studies do not describe the type and duration of drugs used at the time of inclusion and during treatment or how these drugs may affect the experimental and/or standard therapy. Even less information has been published on the potential interaction between concomitant medications and tumor biomarkers. However, we do know that concomitant drugs can complicate cancer clinical trials and biomarker development, thus contributing to their interaction, leading to side effects, and resulting in suboptimal adherence to anticancer treatment. On the basis of these premises and moving from the study by Jurisova *et al.*, which reported the effect of commonly used drugs on the prognosis of women with breast cancer and the detection of circulating tumor cells (CTCs), we comment on the role of CTCs as an emerging diagnostic and prognostic tool for breast cancer. We also report the known and hypothesized mechanisms of CTC interplay with other tumor and blood components, possibly modulated by widespread drugs, including over-the-counter compounds, and discuss the possible implications of commonly used concomitant medications on CTC detection and clearance. After considering all these points, it is conceivable that concomitant drugs are not necessarily a problem, but on the contrary, their virtuous mechanisms can be exploited to reduce tumor spread and enhance the effect of anticancer therapies.

Identifying and isolating circulating tumor cells (CTCs) is the most straightforward method to obtain insight into the progression of a neoplastic disease at a systemic level without using invasive methods. However, today, almost three decades after this brilliant consensus was reached, dealing with CTCs remains complicated by technical difficulties and limited clinical utility^[1]^. An increasing number of studies have been published on CTCs that address all aspects of this biomarker development (technical validity, clinical validity, and clinical utility). In 2004, the United States Food and Drug Administration (FDA) approved CellSearch^TM^ CTC counting for staging patients with metastatic breast, prostate, and colorectal cancers^[2]^. More recently, several cutting-edge studies have reported valuable information obtained from genomic, transcriptomic, and epigenomic analyses on single isolated CTCs^[3]^. However, many uncertainties remain in the CTC domain, including the mechanisms underlying their release, clearance, biological significance (i.e., association with the origin, aggressiveness, and metastatic potential of tumors) in addition to response to treatment.

In their study, Jurisova *et al.* addressed an additional neglected aspect: the impact of concomitant medications on the detection of CTC^[4]^. Concomitant medications include prescription drugs, over-the-counter drugs, or dietary supplements taken by an individual at the time of diagnosis and/or treatment of a specific disease. Patients with cancer are more likely to take concomitant drugs compared with other patients for two main reasons: first, to minimize disease-related symptoms, and second, as an elective remedy for the disease itself, such as in the case of self-prescribed herbal products^[5]^. Concomitant drug use is therefore almost universally observed among patients with cancer, occurring in up to 99% of all cases, whereas it is merely widespread in other types of diseases, ranging between 70% and 83%^[6]^. Concomitant drugs may interact with anticancer drugs and have an effect on outcomes, resulting in erroneous conclusions, which is why the rules of Good Clinical Practice require physicians to pay attention to concomitant drugs used before and during a specific treatment^[7]^.

Because CTCs are used for prognosis as well as for monitoring treatment response, Jurisova *et al.* investigated the possible associations between the use of chronic medications and long-term survival and attempted to avoid the confounding factors caused by the disease itself^[4]^. Importantly, they used a CTC-detection approach that can distinguish CTCs with a frankly epithelial phenotype (referred to as CTC_EP) from those undergoing epithelial-mesenchymal (E/M) transition (EMT) and loss of epithelial protein markers and genes expression (referred to as CTC_EMT). Such a distinction would not have been possible using the FDA-approved CellSearch^TM^ approach, which only enumerates the CTC-expressing epithelial markers (EpCAM and CKs). It should be noted that the EMT is not a binary process. Some cells may achieve a hybrid E/M phenotype or migrate as CTC clusters^[8]^. The ability to attain a hybrid E/M phenotype rather than a full EMT phenotype confers increased metastatic potential, and CTC clusters are observed more frequently in the early stage rather than during the metastatic stage^[9]^. However, long-term treatments, such as anticancer treatments, are known to induce EMT programs, which are often associated with the resistance mechanism, as phenotypic plasticity confers invasiveness and increases the dissemination potential^[10]^. In this case, what is actually a resistance pattern driven by EMT could instead be misinterpreted as a reduction or disappearance of epithelial CTC (and thus considered a treatment response). This phenomenon has been described as “drug-related undetectable CTC status”, and it has been ascribed to the hypoxia-driven EMT triggered by the antineoangiogenic drug bevacizumab^[11]^. Researchers have also described a similar effect in patients treated with the monoclonal antibody denosumab^[12]^.

Luckily, Jurisova *et al.* relied on the detection of both CTC_EP and CTC_EMT in a cohort of > 400 women with stage I-III breast cancer who had complete medical history and information on the use of concomitant medications available^[4]^. Thus, they dodged the problem of misinterpreting the absence of CTC as a sign of absence of systemic dissemination and/or of good prognosis simply owing to the technical failure in detecting CTC_EMT. In addition, 10% of patients had primary tumors lacking hormone receptors, which are basal-like in most cases and rarely express CK19, instead showing high expression of CK5 and CK17^[13]^. Increased CTC_EMT positivity rates were consistently observed for all classes of drugs tested, although none reached statistical significance. In contrast, the use of angiotensin-converting enzyme (ACE) inhibitors was associated with an almost 50% decrease in CTC_EP positivity. The clinical value of interfering with the angiotensin/renin system has rarely been addressed in the literature and has produced controversial results. Specifically, the landmark LACE study reported in the last decade stated that patients with breast cancer undergoing treatment with ACE inhibitors were more likely to experience recurrence compared with nonusers^[14]^. Conversely, another study that investigated the impact of ACE inhibitors in the setting of preoperative treatment reported no immediate effects on tumor response but did find an improvement in relapse-free survival^[15]^. More recently, researchers have suggested that ACE inhibitors have no direct cytotoxicity against tumor cells but rather show the potential to inhibit tumor growth owing to the suppression of vascular endothelial growth factor-induced angiogenesis *in vivo*^[16]^. Thus, we could hypothesize that ACE inhibitors exert an antiangiogenic activity that hinders the extravasation of CTCs and/or the formation of a metastatic colony in distant organs; alternatively, they could induce tumor mass dormancy or prolong dormancy maintenance. Taken together, these findings suggest that medical oncologists should interpret the results of CTCs on the basis of an individual’s use of ACE inhibitors. Furthermore, the findings also have an impact on pooled data collected in clinical studies, suggesting that CTC results should be evaluated separately in users and nonusers of ACE inhibitors.

Drugs other than antihypertensives have been studied with respect to their prognostic effect in cancer, and specifically on the CTCs, which are potential therapeutic targets not only individually but also in aggregated forms with other cell types. For example, in animal models, anti-aggregation therapy can potentially reduce the risk of tumor metastases. Li *et al.* explored genetically modified platelets expressing tumor necrosis factor-related apoptosis-inducing ligand and found that blocking the interactions between platelets and CTCs reduced metastatic potential in a prostate cancer model^[17]^. In addition, platelets expressing tumor necrosis factor-related apoptosis inducing ligand could significantly eradicate cancer cells *in vitro*. Thus, agents that are not strictly anticancer could play a virtuous role in CTC interactions. In fact, clinical evidence has shown that aspirin reduced all-cancer mortality after only 5 years^[18]^. Those results can hardly be interpreted as aspirin only affecting carcinogenesis or early cancer growth and instead suggest that it may act as an inhibitor of cancer metastasis. Finally, treatment with the salicylate diflunisal induced disease remission (complete and partial) in two patients who refused standard treatment. Notably, both responders showed reduced post-treatment CTC levels^[19]^. The most accepted hypothesis is that the downregulation of the platelet-related COX-1 pathway by aspirin may explain the EMT of CTCs and contribute to its antimetastatic effects.

CTCs can also form heteroclusters with neutrophils, and this increases their metastatic potential^[20]^. Preclinical studies in mouse models have demonstrated that the anti-inflammatory zileuton, an inhibitor of arachidonate 5-lipoxygenase (5-LOX) and thus of leukotriene production, reduces spontaneous metastasis^[21]^; more recently, zileuton-loaded nanoparticles have been shown to reduce breast CTCs^[22]^. Interestingly, among 5-LOX inhibitors, there is curcumin, which is often used as a food supplement in general as well as in cancer patient populations^[23]^. However, Jurisova *et al.* did not observe any effects of nonsteroidal anti-inflammatory drugs, probably because of the low number of patients reporting the regular use of such medications^[4]^. All of these data are limited by the low number of patients and are, in some cases, purely anecdotal. Nevertheless, they offer the opportunity to hypothesize that CTCs not only may be used as a surrogate marker of tumor response but also may themselves become a treatment target with therapies aimed at decreasing their survival in the circulation and tissue homing.

Similarly to the CTC/platelets and CTC/neutrophils heterogroups, CTC clusters are central to metastatic potential^[24]^. Preclinical data showing that the Na^+^/K^+^-ATPase inhibitors digoxin and ouabain used as antiarrhythmic agents are able to disaggregate CTCs clusters^[25]^ led to the ongoing “Digoxin Induced Dissolution of CTC Clusters” trial (NCT03928210), the results of which are urgently awaited.

Studies such as the one reported by Jurisova *et al.* contribute to the CTC field because even slight effects on CTC enumeration can hide interesting mechanisms exerted by common drugs that can be a premise to their repurposing or off-target use^[4]^.

## DECLARATIONS

### Copyright

© The Author(s) 2023.
